# Layer-by-layer coated hybrid nanoparticles with pH-sensitivity for drug delivery to treat acute lung infection

**DOI:** 10.1080/10717544.2021.2000676

**Published:** 2021-11-12

**Authors:** Ji Luo, Xiaobo Li, Siyuan Dong, Peiyao Zhu, Wenke Liu, Shuguang Zhang, Jiang Du

**Affiliations:** Department of Thoracic Surgery, The First Affiliated Hospital of China Medical University, Shenyang, China

**Keywords:** Layer-by-layer, hybrid NP, pH-responsive, antimicrobial, ALI

## Abstract

Bacteria-induced acute lung infection (ALI) is a severe burden to human health, which could cause acute respiratory distress syndrome (ARDS) and kill the patient rapidly. Therefore, it is of great significance to develop effective nanomedicine and therapeutic approach to eliminate the invading bacteria in the lung and manage ALI. In this study, we design a layer-by-layer (LbL) liposome-polymer hybrid nanoparticle (HNP) with a pH-triggered drug release profile to deliver antibiotics for the eradication of bacteria to treat ALI. The liposome is prepared by the lipid film hydration method with a homogenous hydrodynamic diameter and low polydispersity index (PDI). The antibiotic spectinomycin is efficiently loaded into the liposomal core through the pH-gradient method. The pH-sensitive polycationic polymer poly(*β*-amino ester) (PBAE) and polyanionic sodium alginate (NaAIg) layers are decorated on the surface of liposome in sequence *via* electrostatic interaction, resulting in spectinomycin-loaded layer-by-layer hybrid nanoparticles (denoted as Spe@HNPs) which have reasonable particle size, high stability, prolonged circulation time, and pH-triggered drug release profile. The *in vitro* results demonstrate that Spe@HNPs can efficiently induce the death of bacteria with low minimum inhibitory concentration (MIC) against *Staphylococcus aureus* (*S. aureus*) and drug-resistant MRSA BAA40 strains. The *in vivo* results reveal that Spe@HNPs can eradicate the invading MRSA BAA40 with improved antimicrobial efficacy and low side-effect for ALI treatment. This study not only reports a promising nanomedicine but also provides an effective method to prepare nanoplatforms for drug delivery and controlled release.

## 1. Introduction

Bacteria-induced infectious diseases are a severe burden to human health in the world (Prestinaci et al., 2015; van der Poll et al., 2017). The discovery of antibiotics is supposed to be a therapeutic strategy in the treatment of these infectious diseases in the past several decades (Arias and Murray, 2015; Ling et al., 2015), but these small molecule drugs are easily and predominately eliminated by the reticuloendothelial system (RES) like kidney, resulting in poor antibacterial efficacy and low bioavailability (Roberts, 2011; Kuno et al., 2016). Furthermore, the off-targeting of the antibiotics may cause severe side-effects, particularly attacking the healthy tissue and organ, and leading to susceptibility to infections (Dinarello, 2010; Wade and Williams, 2019). In addition, drug-resistance of ‘superbugs’ has been emerged and raised as one of the biggest threats to the antimicrobial battleground, resulting from the misuse/overuse of antibiotics in humans and animals (Holmes et al., 2016; Thanner et al., 2016; Ayukekbong et al., 2017; Blaskovich, 2018; Tacconelli et al., 2018). These drug-resistant bacteria can survive with the common free antibiotic treatment, resulting in longer hospital stays with high costs and high mortality rates (Aslam et al., 2018). For instance, spectinomycin/methicillin-resistant *Staphylococcus aureus* is able to cause severe infections on the skin and some soft tissues in the body. And this superbug is also associated with some acute infectious diseases such as nosocomial pneumonia. As reported, drug-resistant bacteria cause more than 20,000 deaths per year in the United States (Klein et al., 2007; Klevens et al., 2007; Mendy et al., 2016; Kavanagh, 2019). Therefore, it’s of great importance and urgency to develop effective therapeutics and treatment strategies to eliminate the invading bacteria for anti-infection, especially ALI treatment.

With the rapid development of nano-bio-technology, multi-functional biomaterials-based drug delivery systems have attracted more and more attention for improving therapeutic efficacy (Radovic-Moreno et al., 2012; Gupta et al., 2019; Yang et al., 2020). For instance, Wang and coworkers developed an infectious microenvironment-sensitive nanoparticle for simultaneous delivery of ciprofloxacin and TPCA-1 (anti-inflammatory drug) to manage the bacteria-caused ALI and sepsis in mice (Zhang et al., 2018). Ruoslahti and colleagues reported the biocompatible nanoparticles which could target the invading *S. aureus* in the body, showing high antimicrobial activity and low systemic side-effects to treat the skin and lung infection (Hussain et al., 2018).

**Figure 1.**
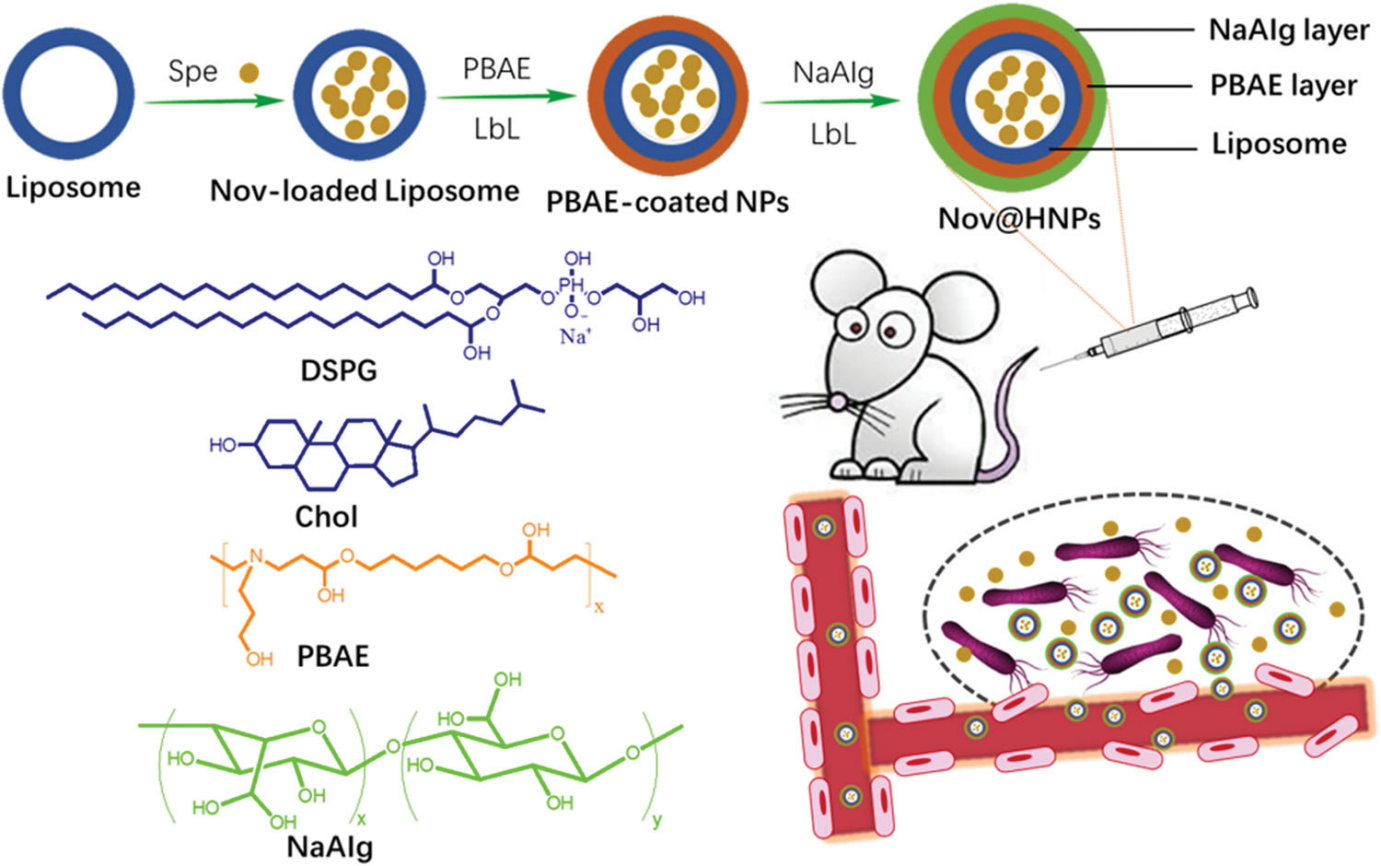
Illustration for the preparation of pH-responsive Spe@HNPs with layer-by-layer structure and drug delivery for acute lung infection treatment.

In this study, inspired by the acidic microenvironment at the infection site (Zhang et al., 2019; Ma et al., 2020; Chen et al., 2021), we developed a hybrid nanoparticle based on liposome and polymer through an extrusion and LbL processes for delivery of antibiotics to treat the acute lung infection (Figure 1). Here, spectinomycin is selected as a model antibiotic (Lee et al., 2014), and was encapsulated into the liposomal core through the pH-gradient method. The liposomes were prepared based on 1, 2-distearoyl-*sn*-glycero-3-phospho-(1′-*rac*-glycerol) (DSPG) and hydrophobic cholesterol (Chol) with good biocompatibility. The polycationic polymer poly(*β*-amino ester) (PBAE), widely used as drug delivery carriers with pH sensitivity, was used as a functional layer for pH-triggered drug release performance (Zhang et al., 2014; Kaczmarek et al., 2016; Huang et al., 2017; Li et al., 2018; Men et al., 2020). The polyanionic sodium alginate (NaAIg) layer is successively deposited on the surface of NPs *via* the LbL process (Jain and Bar-Shalom, 2014; Ilgin et al., 2020), resulting in Spe-loaded liposome-polymer hybrid NPs (Spe@HNPs). The physicochemical properties of Spe@HNPs, including hydrodynamic diameter, surface charge, drug loading content, and release performance are thoroughly investigated. The antibacterial efficacy and cytotoxicity *in vitro* are assessed. The therapeutic efficacy against acute lung infection *in vivo* is evaluated. This designed Spe@HNPs might be a promising nanomedicine for anti-infection.

## 2. Materials and methods

### 2.1. Materials

Lipid 1, 2-distearoyl-*sn*-glycero-3-phospho-(1′-*rac*-glycerol) (DSPG) and hydrophobic cholesterol (Chol) were purchased from Avanti Polar Lipids. Spectinomycin (≥95%), sodium alginate (NaAIg), thiazolyl blue tetrazolium bromide (MTT, 98%) were purchased from Sigma Aldrich. Chloroform, dimethyl sulfoxide (DMSO), and other organic solvents were analytical grade and pursued from Sigma Aldrich. *S. aureus* (*S. aureus*, ATCC 29213) and *S. aureus* (MRSA BAA40) strains, standard NIH 3T3 mouse fibroblast cells, and mediums were purchased from InVivos.

### 2.2. Preparation of Spe-loaded liposome

Spe-loaded liposomes were successfully prepared according to the previous reports (Deng et al., 2013; Freag et al., 2016; Mensah et al., 2019; Men et al., 2020). Briefly, DSPG and Chol at a mass ratio of 3: 1 were dissolved in a mixed solvent (chloroform: methanol: water= 60: 32: 8, v/v) in a round-bottom flask. A thin lipid film was prepared using rotary evaporation. The temperature was 40 °C and the press was 150 mbar. After the solvent was completely removal, the resulted film was mixed with citric acid buffer (pH 4.0) at 65 °C with sonication for 90 min, followed by filtering with 100 nm PES syringe filter. Then, the sodium carbonate buffer was dropped to increase the pH of the liposomal suspension to about 6.8. The model drug Spe at different feed ratios was then added to load through the pH gradient method. Then, the solution was purified using centrifugal filtration for three-time. The Spe-loaded liposomes were obtained and stored for the study.

### 2.3. Preparation of spe@HNPs

The Spe@HNPs were prepared according to the references (Deshmukh et al., 2013; Morton et al., 2013; Men et al., 2020). In brief, 5 mg of PBAE was added into 2 mg of Spe-loaded liposomes solution (2 mL), resulting in the mixed solution which was incubated at room temperature with sonication for about 5 s. After centrifugation at 2000 g for 30 min, the PBAE-coated Spe-loaded NPs were obtained. Similarly, 5 mg of NaAIg was added into the solution, and NaAIg layer was successfully coated on the surface, resulting in the Spe@HNPs. The particle size, polydispersity index (PDI), and surface charge of samples after each step were measured to validate the successful each layer deposition.

### 2.4. Characterization

The particle size and surface charge of Spe-loaded liposomes, Spe-loaded NPs coated with PBAE layer and bilayered Spe@HNPs were measured using dynamic light scattering (DLS, Malvern Zetasizer Nano S, Malvern, UK). The sample was re-suspended in PBS, and measured in a quartz cuvette (1.0 mL) at room temperature. In order to evaluate the stability of the system, the particle size and PDI of samples were recorded after incubation in the serum solution. 1 mg of Spe@HNPs solution in PBS (1 mL) containing 20% fetal bovine serum (FBS) at pH 7.4 was prepared first. Then, the hybrid nanoparticle solution was kept in an incubator at 37 °C with 110 rpm for five days. At predetermined time intervals, the particle size and PDI of the sample were recorded using DLS. Moreover, to further confirm the stability of the system, 2 mg of Spe@HNPs were re-suspended in PBS (1 mL, pH 7.4) or 5% glucose solution. Then, the original solution was diluted at 1/10, 1/100, and 1/1000 to prepare the samples for DLS measurement.

In order to confirm the pH sensitivity of Spe@HNPs, 1 mg of sample in PBS (1 mL) at different pH conditions were prepared firstly. After incubation with 110 rpm at 37 °C, the hydrodynamic diameter, polydispersity index, and surface charge of solution were monitored using DSL as aforementioned.

The morphology of the sample was determined by field-emission scanning electron microscopy (FE-SEM, JEOL JSM 6701 F). The Spe@HNPs in PBS were centrifuged at 7000 rpm for 10 mins with a pellet which was resuspended in deionized water. 2.5 µL of sample suspension was dropped on the copper foil and dried overnight. Then, the Spe@HNPs were coated with platinum with current 10 A for 120 s. The images were then taken under FE-SEM at an acceleration voltage of 5 kV

### 2.5. Drug loading capacity

To evaluate the drug loading efficacy of HNPs, the drug loading content (LC) and encapsulated efficiency (EE) of Spe@HNPs were confirmed by high-performance liquid chromatography (HPLC). In brief, Spe@HNPs (0.5 mL, 2 mg/mL) was added into DMSO (10 mL) with stirring at room temperature for 1 h. The sample was measured using HPLC, and the amount of Spe was calculated based on the standard curve. The LC of Spe@HNPs was the weight ratio of Spe loaded in the HNPs to the total Spe@HNPs sample. The EE was the weight ratio of Spe loaded in the HNPs to the total Spe drug when preparing the Spe@HNPs.

### 2.6. Spe release profiles from spe@HNPs

The Spe release performance *in vitro* from Spe@HNPs was studied using the dialysis method ( Zhang et al., 2019; Men et al., 2020). Briefly, Spe@HNPs (2 mL, 2 mg/mL) was re-suspended into PBS (4 mL) at pH of 7.4 or 6.0, followed by transferring the solution into a cellulose dialysis bag (molecular weight cutoff, MWCO 3500-4000). Then, the dialysis bag was immersed into the according to PBS (44 mL, pH 7.4 or 6.0) in a beaker with stirring 110 rpm at 37 °C. At the predetermined time, the sample solution (2 mL) was taken outside of the dialysis bag for HPLC measurement, and the same volume of fresh PBS (pH 7.4 or 6.0) was added into the solution. The percentage of accumulative drug release (*E*_r_) from Spe@HNPs was calculated according to the equation:

$$E_{r}=\frac{V_{e}\sum_{1}^{n-1}C_{i}+V_{0}C_{n}}{M_{Spe}}\times 100\%$$

where, *m*_Spe_ is the mass weight of Spe loaded into the HNPs, *V*_e_ is the volume of PBS in the dialysis bag (6 mL), *V*_0_ is the total volume of PBS (50 mL), and *C*_i_ is the concentration of release Spe for the *i*_th_ sample.

### 2.7. Cell culture

NIH 3T3 cells were cultured in the prepared Dulbecco's modified eagle medium (DMEM) which were added with 10% FBS, 100 units/mL penicillin, and 100 μg/mL streptomycin. NIH 3T3 cells were saved at 37 °C in an incubator with 5% CO_2_.

### 2.8. Cytotoxicity assay

MTT assay was utilized to study the toxic effect of free Spe, blank HNPs, and Spe@HNPs against NIH 3T3 cells. In brief, the NIH 3T3 cells were cultured in the prepared DMEM, and were collected at the logarithmic growth phase. The cells were seeded into 96-well plates at a concentration of 5000 cells/well in 200 μL. After incubation at 37 °C overnight, the medium was removed. 200 μL of samples (free Spe, HNPs, and Spe@HNPs) at different conditions in DMEM was added into every well. The fresh medium was used as a control and added into the well similarly. The 96-well plates containing samples and cells were incubated in the incubator for 24 h. Then, the solution was removed, followed by adding MTT solution (200 μL/well, 1 mg/mL). After that, the 96-well plates were shaken at 150-200 rpm for 5-10 min. The plates were further incubated for another 4 h, and the medium was discarded. Then, DMSO (200 μL) was added with stirring at 150 rpm for 10-15 min. Finally, the plates were read with a microplate reader at 570 nm. The cell viability was calculated according to the equation:

$$Cell\;\;viability\;\;(100\;\%)=\frac{A_{sample}-A_{blank}}{A_{control}-A_{blank}}\times \;\;100\;\;\%$$

where, *A*_control_ and *A*_sample_ are the absorbances at 570 nm with or without the sample treatment. *A*_blank_ was the absorbance at 570 nm only with the medium.

### 2.9. In vitro antimicrobial efficacy against bacteria

Free Spe and Spe@HNPs were diluted in 5 mM HEPES at a concentration of 2 mg/mL. Compounds were dispensed in the first wells of a flat bottom 96-wells microtiter plate and serially two-fold diluted with Mueller Hinton Broth (MHB) into successive wells in a final volume of 100 μL. Bacteria were cultured at 37 °C in MHB in a mid-log phase. 100 μL of diluted bacteria were dispensed in each well of the plates. 100 μL of bacteria were inoculated in MHB only and used as bacterial growth-controls. Plates were sealed with parafilm and incubated at 37 °C for 18 h. The minimal inhibitory concentration (MIC) was measured using the absorbance at 600 nm with a microplate reader (Liao et al., 2019; Si et al., 2021).

### 2.10. Mice

Adult CD-1 (18-20 g) mice were saved in polyethylene cages with stainless steel lids at 20 °C-22 °C with a 12 h light/dark cycle. The cages were covered with a filter cap. These mice were fed with food and water *ad-lib*. The China Medical University Institutional Animal Care and Use Committee approved all animal care and experimental protocols used in the studies.

### 2.11. Antimicrobial efficacy in vivo

The mice were anesthetized using intraperitoneal (*i.p.*) injection of ketamine (120 mg/kg) and xylazine (6 mg/kg) mixture in saline. Then, the mice were placed in a supine position head up on aboard. Afterward, the trachea of a mouse was exposed, and 10^6^ CFU of MRSA BAA40 per mouse was intratracheally administrated. The mouse was held upright for 1 min after administration. 4 hours later, the mice were grouped randomly and intravenously injected with PBS, HNPs (4 mg/kg), free Spe (4 mg/kg), Spe@HNPs (equal to 4 mg/kg of free Spe), respectively. At 24 h, the mice were anesthetized, and the trachea was cannulated. A needle was inserted into the cannulated trachea, and PBS (1.5 mL-3 mL) was infused and withdrawn to collect the lung bronchoalveolar lavage fluid (BALF) which was stored for future analysis.

### 2.12. Measurement

BALF was centrifuged at 350 g for 5 min, and the supernatant was collected. The CFU in BALF was measured using LB plates. The certain volumes of the supernatant were added to the plates and incubated at 37 °C for 16-20 hours. Then, the CFU numbers were counted. The concentrations of inflammatory factors TNF-*α*, IL-6, and IL-1*β* in the supernatant were determined with ELISA MAX Deluxe Sets (Biolegend, San Diego, CA). The protein contents in the supernatant of BALF were determined with the BCA method using a commercial kit (Thermo Scientific, Rockford, IL). The pellet was collected and counted to record the leukocytes number.

### 2.13 H&E Staining

The lungs were harvested after different treatments (PBS, HNPs, free Spe, and Spe@HNPs). Then, the lungs were fixed with 10% formalin, embedded in paraffin and sectioned at 5 μm, followed by staining with hematoxylin and eosin. The prepared slices were imaged by fluorescence confocal microscopy (ZEISS, Observer. Z1, USA).

### 2.14. Statistical analysis

The experimental data were presented with an average value, expressed as mean ± standard deviation (s.d.). Statistical analysis was conducted using one-way ANOVA or Student's *t*-test of Origin 8.5.

## 3. Results and discussion

### 3.1. Preparation and characterization of Spe@HNPs

**Figure 2.**
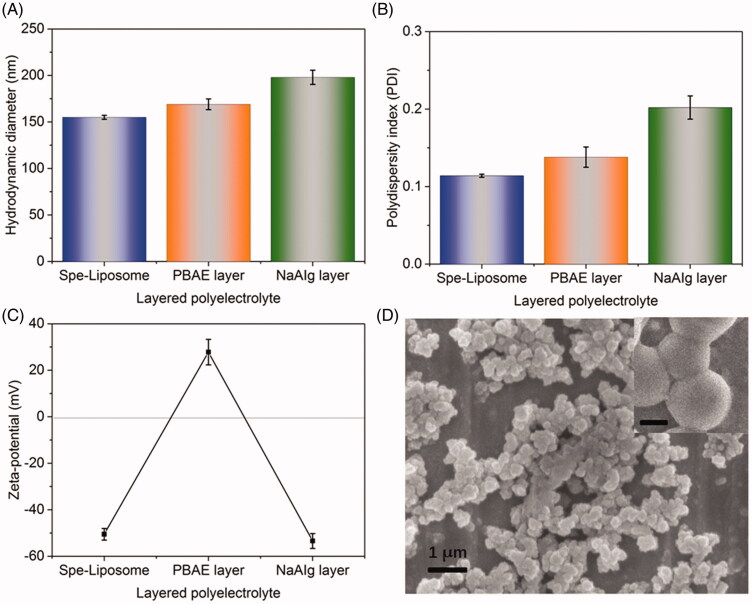
Characterization of Spe@HNPs system. Particle size (A), polydispersity index (PDI, B), and charge reversal in zeta-potential (C) of Spe-loaded liposomes, single-layered NPs, and Spe@NPs. The data are shown as mean ± s.d. (*n* = 3 independent experiments). (D) SEM image of Spe@NPs (insert: scale bar: 100 nm).

The bilayered drug-loaded liposome-polymer hybrid nanoparticles (Spe@HNPs) were prepared using the film hydration method, extrusion, and layer-by-layer processes. Firstly, the liposomes were prepared based on DSPG and Chol at a mass ratio of 3:1, followed by loading the drug *via* the pH-gradient method. The pH in the liposomal core was about 4.0, while the pH outside was adjusted to about 6.5. The solubility of the drug was significantly increased at pH 4.0 compared with that at pH 6.5 due to the protonation of amine residues in the spectinomycin. Then, the PBAE layer and NaAIg layers were successively deposited on the surface of Spe-loaded liposomes *via* the LbL process through the polyelectronic interaction. This process was recorded by measurement of hydrodynamic diameter, PDI and zeta-potential of the system after each deposition of a layer, as shown in Figure 2(A–C). The particle size of Spe-loaded liposomes was approximately 150 nm. And the size was increased to about 170 nm after deposition of the PBAE layer. After the deposition of the anionic NaAIg layer, the particle size increased to 198 nm (Figure 2(A)). The sustaining increase of particle size of the sample after each layer deposition suggested that the PBAE and NaAIg layers were successively coated on the surface of nanoparticles. Figure 2(B) showed that the PDI values of samples were increased from 0.114 to 0.202 (< 0.3) after deposition of functional layers, showing that the drug-loaded samples before and after the LbL process had good uniformity. To further confirm the successful deposition of each functional layer, the surface charge of samples at each step was recorded and shown in Figure 2(C). The zeta-potential of Spe-loaded liposomes without coating was about −50.5 mV (negative charge), while it was significantly increased to +27.8 mV (positive charge) after coating of polycationic PBAE layer. After deposition of polyanionic NaAIg layer, the surface charge of Spe@HNPs was obviously decreased to about −55.0 mV (negative charge) again. Additionally, the characteristic peak at 1680 cm^−1^ was from the stretching vibration of amides in PAE, suggesting the successful deposition of the PBAE layer on the surface of liposomes. The characteristic peaks from 3200 cm^−1^ to 1680 cm^−1^ were attributed to the intermolecular hydrogen bonding and muti-molecule association, in DSPG, Chol, PBAE, and NaAIg, suggesting the successful deposition of the NaAIg layer on the surface of liposomes (Figure S1). In summary, the complete charge reversal (negative-positive-negative) after each deposition of functional layer and FT-IR spectra of samples indicated that PBAE and NaAIg layers were successfully coated on the drug-loaded liposomes. Next, the morphology of Spe@HNPs was characterized using FE-SEM, as shown in Figure 2(D). The Spe@HNPs exhibited uniformly spherical in shape with a reasonable particle size which was consistent with the result of DLS measurement. The size was slightly decreased, resulting from the lyophilization process for the FE-SEM test. Taken together, the bilayered multifunctional Spe@HNPs based on liposomes, PBAE and NaAIg layers were successfully prepared using the LbL process. Then, the drug loading capacity of hybrid nanoparticles was next evaluated. The drug loading contents and encapsulation efficiency of Spe@HNPs at different ratios of liposome to the drug were listed in Table 1. At the mass ratio of 1: 1 (Spe: liposome, m/m), the LC was 335.7 µg of drug per 1 mg liposome, and EE was about 33.0%. When the ratio in feed was increased to 2: 1, the LC was increased to 478.3 µg/mg, while the EE was decreased to 24.4% because of the excess unloaded drug. With the increase of drug in feed (3: 1), although the LC was slightly increased, the EE was lower than 20%. Therefore, the Spe@HNPs with the mass ratio of Spe: liposomes= 2: 1 were used for the following studies in this work.

**Table 1.** Spe loading capacity of Spe@NPs at different mass ratios.

Liposomes (mg)	Spe (mg)	LC (µg/mg)	EE
10	10	335.7	33.0
10	20	488.3	24.4
10	30	510.5	16.9

### 3.2. PH-sensitivity and stability

**Figure 3.**
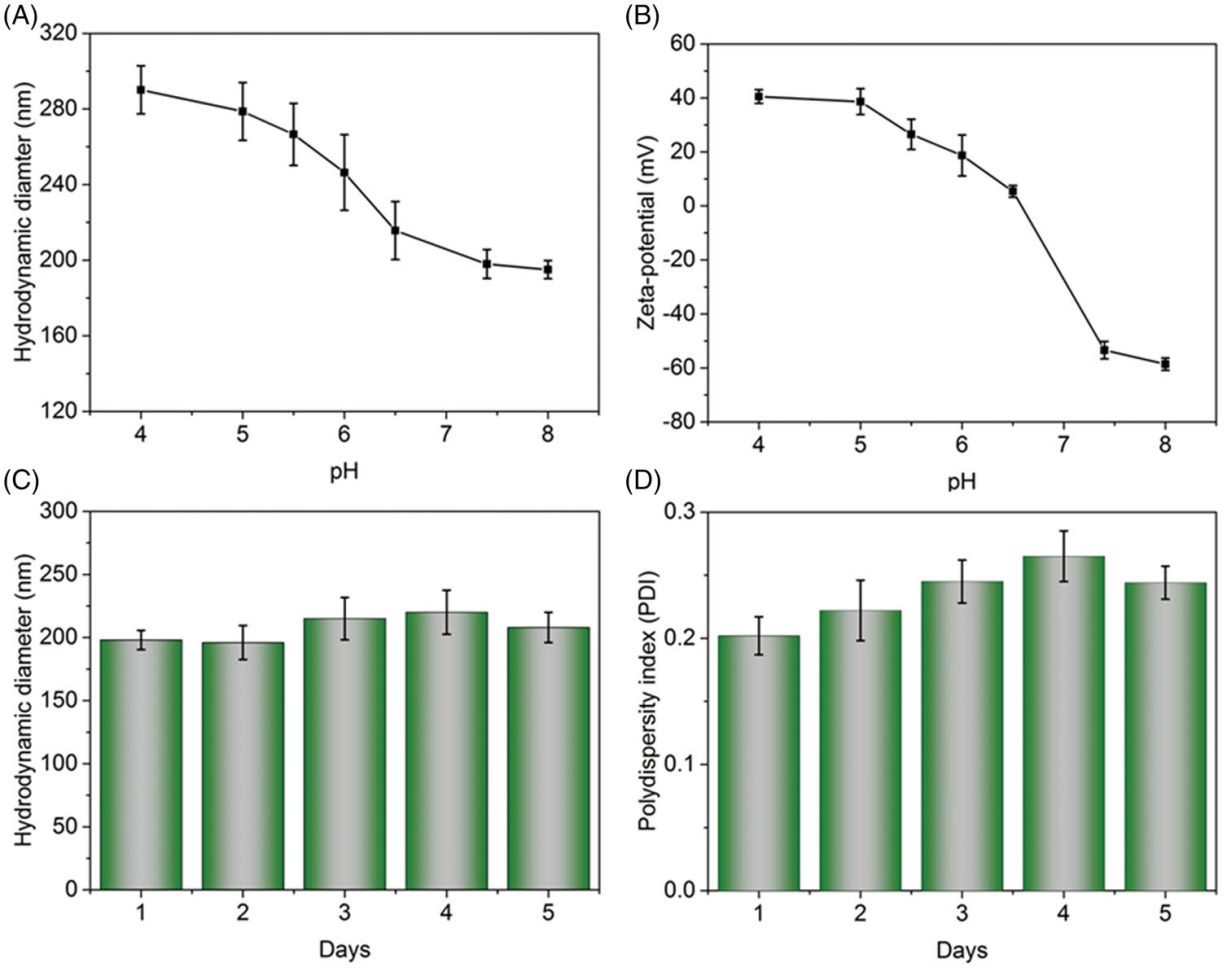
Evaluation of pH-responsive property and stability of Spe@NPs system. The hydrodynamic diameters (A) and zeta-potential (B) of Spe@HNPs at different conditions. Hydrodynamic diameters (C) and PDI (D) of Spe@HNPs after incubation in PBS (pH 7.4) with 20% FBS at 37 °C for 5 days. The data are shown as mean ± s.d. (*n* = 3 independent experiments).

To analyze the pH-responsive property of Spe@HNPs, the hydrodynamic diameter, PDI and surface charge of Spe@HNPs at different pH conditions were measured, as shown in Figure 3(A,B) and Figure S2. The particle size of Spe@HNPs was dramatically increased from about 200 nm to 300 nm with the decrease of pH from 8.0 to 4.0, especially in weakly acidic conditions (Figure 3(A)). The PDI of Spe@HNPs displayed similar change trends, especially the change at the pH range of 7.0 to 6.0 (Figure S2). The reason might be that the protonation of tertiary amine residues in the PBAE layer an acidic environment transferred the solubility of PBAE from hydrophobicity to hydrophilicity, leading to the swelling of Spe@HNPs that resulted in the increase of particle size. Furthermore, the surface charge of Spe@HNPs was increased from negative to positive, due to the ionization of tertiary amine residues in the PBAE layer (Figure 3(B)). Collectively, the changes of particle size, PDI, and surface charge of Spe@HNPs dependent on the different pH proved that this bilayered system Spe@HNPs showed pH-sensitivity.

The high stability of the drug delivery system is the precondition for clinic use. Herein, the serum stability of Spe@HNPs was evaluated. Firstly, the particle size and PDI of Spe@HNPs after incubation in PBS (pH 7.4) with 20% FBS at 37 °C were recorded every day, as shown in Figure 3(C,D). The particle size of Spe@HNPs was slightly increased from 200 nm to about 220 nm after 5-day incubation, suggesting the system had high serum stability. Additionally, the PDI values of Spe@HNPs were still less than 0.3 at 5 days. There was no significant increase in size and PDI. To further evaluate the stability of Spe@HNPs, the particle sizes and PDI values of Spe@HNPs in PBS or 5% glucose solution were measured, as shown in Figure S3. No significant changes in particle size and PDI were observed after dilution by 1000-time, indicating that Spe@HNPs had high stability. All these findings demonstrated that the prepared Spe@HNPs had high serum stability, suggesting that this system could have a prolonged circulation time in the body which facilitated the accumulation of Spe@HNPs at the infection site. In summary, the bilayered system Spe@HNPs exhibited reasonable pH-sensitivity and high stability which might be used for drug delivery with a pH-triggered drug release profile.

### 3.3. *In vitro* pH-triggered drug release performance

**Figure 4.**
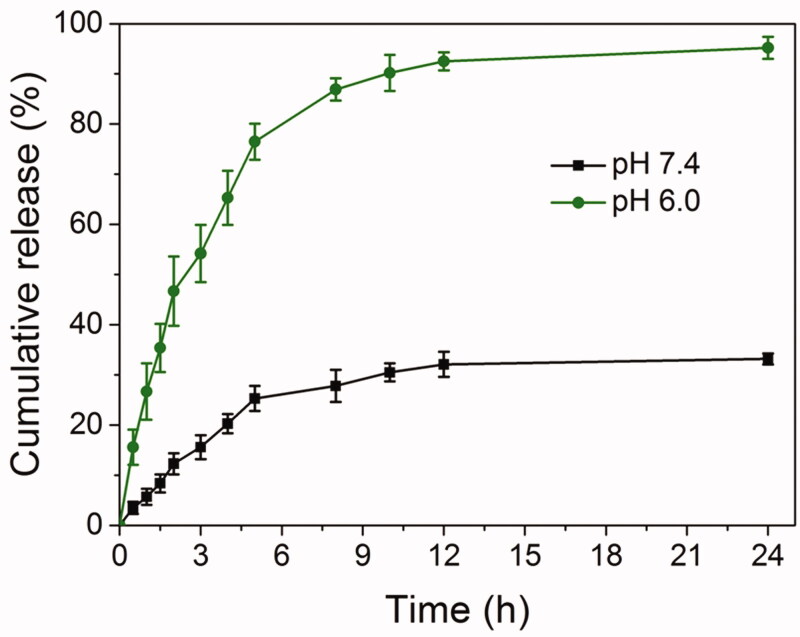
*In vitro* pH-triggered drug release performance of Spe@HNPs at pH 7.4 and 6.0. The data are shown as mean ± s.d. (*n* = 3 independent experiments).

We next studied the drug release profiles of Spe@HNPs in PBS (pH 7.4, normal physiological condition) and weakly acidic buffer solution (pH 6.0, infectious microenvironment), as shown in Figure 4. The drug release rate and accumulative release amount of Spe@HNPs at different pH conditions were obviously different as seen from the results. At pH 7.4, the drug release rate was slow, and the accumulative drug release amount was less than 30% for 10 h and about 33% for 24 h. At normal physiological conditions, the tertiary amine residues in PBAE were not ionized, and Spe@HNPs were compact. The drug molecules were protected well in the liposomes. In contrast, the drug release rate and accumulative release amount of Spe from Spe@HNPs were dramatically accelerated at pH 6.0. About 90% of the loaded drug was released from HNPs at 10 h, and almost all of the drug was released at 24 h. The reason could be that the tertiary amine residues in PBAE were fully protonated at pH 6.0 which led to the swelling of HNPs, resulting in a rapid drug release rate and enhanced accumulative release amount. In addition, the acidic external environment also facilitated the drug release in comparison to normal physiological conditions. The drug release performance of Spe@HNPs also suggested that the HNPs were pH-sensitive which was consistent with the results in Figure 3. In summary, the prepared Spe@HNPs showed pH-triggered drug release profiles, and acid could significantly enhance the drug release rate and accumulative release amount. This specific property could be used for drug-controlled release on-demand.

### 3.4. Antimicrobial efficacy in vitro

Next, the antimicrobial efficacy of Spe@HNPs against *S. aureus* and drug-resistant bacterium MRSA BAA40 was evaluated, as shown in Table 2. The MIC values of free Spe for *S. aureus* were approximately 2 µg/mL, while the MIC of Spe@HNPs was less than 1 µg/mL. This result displayed that both free Spe and Spe@HNPs showed a high antimicrobial effect for *S. aureus*. However, for drug-resistant bacterium MRSA BAA40, the MIC of free Spe was not found (higher than 64 µg/mL), indicating the low antimicrobial activity and poor inhibition effect for MRSA BAA40. By contrast, Spe@HNPs still showed high antimicrobial activity with much lower MIC (4 µg/mL). Moreover, we completed the time-killing assay experiment (Figure S4) to further evaluate the antimicrobial efficacy of Spe@HNPs *in vitro*. The results showed that both free Spe and Spe@HNPs could efficiently inhibit the growth of *S. aureus* compared with control. However, for MRSA BAA40, free Spe exhibited a negligible inhibition effect. By contrast, the Spe@HNPs can obviously inhibit the MRSA BAA40 compared with free Spe and control, showing the high antimicrobial efficacy of Spe@HNPs. The reason could be that the positively charged Spe@HNPs at weekly acidic conditions can interrupt the cytoplasmic membrane and cause the leakage of cytosol, followed by facilitating the pharmaceutical effect of formulation, resulting in the death of bacteria. This synergistic effect significantly improved the antimicrobial efficacy of Spe@HNPs. In order to satisfy the requirement of the biomedical application, the system should have a negligible toxic effect. Therefore, the *in vitro* cytotoxicity of free Spe, HNPs, and Spe@HNPs against NIH 3T3 cells was measured, as shown in Figure S5. The cytotoxicity of HNPs was slightly increased with the increase of concentration. The cell viability was still approximately 90% even at the highest concentration of 500 µg/mL, indicating the negligible toxic effect of blank HNPs. For free Spe, the cell viability of NIH 3T3 was obviously decreased with the concentration increase. The cell viability was about 80% when the concentration of free Spe was 100 µg/mL. Less than 50% of cells were alive when the concentration was higher than 300 µg/mL. In contrast, the cytotoxicity of Spe was obviously reduced after formulation in Spe@HNPs. At the highest concentration of 500 µg/mL, the cell viability was still higher than 80%. Summarily, the prepared Spe@HNPs could effectively induce the death of drug-resistant bacterium with negligible cytotoxicity.

**Figure 5.**
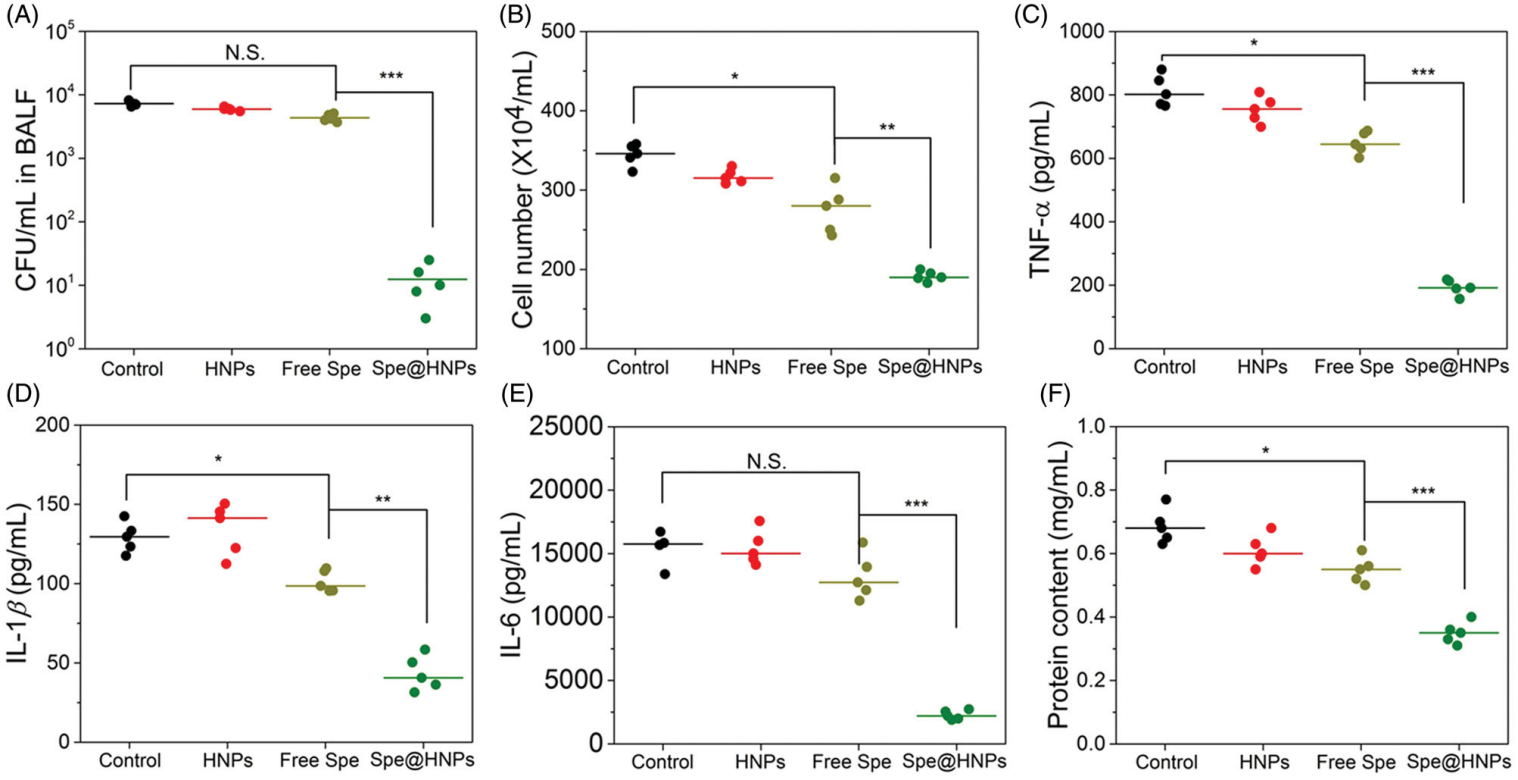
Therapeutic efficacy of Spe@HNPs for an acute lung infection. CFU of bacteria (A), leukocyte number (B), protein content (C), TNF-α (D), IL-1β (E), and IL-6 (F) in BALF of mice infected by MRSA BAA40 20 h after the treatments with PBS, HNPs, free drug or Spe@HNPs. The data are shown as mean ± s.d. (*n* = 5 independent experiments). *P* values: *p < .05, **p < .01, ***p < .001, N.S. (no significant difference) *p* > .05.

**Table 2.** Summary of MIC of samples against *S. aureus* and MRSA BAA40.

Sample	Minimum inhibitory concentration (MIC, µg/mL)	
	*S. aureus*	MRSA BAA40
Free Spe	2	>64
Spe@HNPs	<1	4

### 3.5. Therapeutic efficacy of Spe@HNPs for ALI

Next, we investigated whether the Spe@HNPs could eliminate the bacteria after MRSA BAA40 was directly administrated to the mouse lung. At 4 h post-administration of bacteria into the lung, free Spe, HNPs, and Spe@HNPs were intravenously (*i.v.*) injected into the ALI-bearing mice. At 24 h, the BALF was collected and analyzed to evaluate the therapeutic efficacy, as shown in Figure 5. The CFU in BALF of mice treated with Spe@HNPs was remarkably decreased compared with free Spe treatment and controls (Figure 5(A)), demonstrating that the bacterial proliferation was effectively inhibited by systemic administration of Spe@HNPs. The number of infiltrated leukocytes in BALF was also recorded after different treatments (Figure 5(B)). The free Spe treatment could slightly remit the infiltration of leukocytes, while the Spe@HNPs treatment was able to sharply reduce the number of infiltrated leukocytes. Moreover, the histological studies of lungs after Spe@HNPs treatment for 20 h (Figure S6) displayed that the leukocyte infiltration was obviously decreased compared with other controls, indicating the reduced inflammation level. Furthermore, the inflammatory factors (TNF-*α*, IL-1*β*, and IL-6, Figure 5(C–E)) were obviously decreased after Spe@HNPs treatment in comparison to others. These results proved that the inflammation of mice treated with Spe@HNPs was well mitigated, showing the reduction of bacterial burden in the lungs. As reported, the protein permeability in the lung was associated with the vasculature integrity, and the low vasculature integrity suggested severe inflammation (Mehta and Malik, 2006; Molinaro et al., 2016; Zhang et al., 2018). As shown in Figure 5(F), the protein content in BALF of mice treated with Spe@HNPs was much lower compared with free Spe and other treatments, indicating that the lung vasculature was repaired after the invasive bacteria were removed. In addition, the blank carrier HNPs also exhibited a slightly therapeutic effect compared with control, possibly due to the positive charge on the surface of NPs which broke the bacterial membrane and induced the death of bacteria. Furthermore, the plasma Spe concentration as a function of time was examined by intravenous injection (*i.v.*) of various formulations to healthy mice. Figure S7 showed the pharmacokinetics (PK) of free Spe and Spe@HNPs *in vivo*. The blood half-life (*t*_1/2_) of free Spe was less than 0.5 h, showing the free Spe molecules were rapidly cleared from the blood which might lead to poor therapeutic efficacy. In contrast, Spe@HNPs had prolonged blood circulation time (*t*_1/2_ = 5 h) due to the protection of HNPs, indicating the enhanced accumulation of system and high concentration of Spe in the lung which would lead to the higher therapeutic efficacy. The biosafety of Spe@HNPs was preliminarily evaluated here to prove the potential use in the clinic, as shown in Figures S8 and S9. The results of blood biochemistry analysis (Figure S8) indicated that the heart function marker (CK), hepatic function markers (ALT, AST), and renal function markers (CREA, BUN) in the Spe@HNPs group exhibited negligible difference compared with the normal group. The weight of major organs (especially lung) treated with free Spe@HNPs showed no difference compared with the normal group. However, the weight of lungs of mice treated with free Spe was significantly decreased in comparison to those of normal and Spe@HNPs groups. These results suggested the high biosafety of Spe@HNPs with high therapeutic efficacy. Taken together, the prepared Spe@HNPs could remarkably improve the therapeutic efficacy for a drug-resistant bacterium-induced acute lung infection and mitigate the inflammation response with reduced side-effect.

## 4. Conclusion

In summary, we have successfully developed a pH-responsive drug delivery system (Spe@HNPs) composition of liposomes loaded with antibiotics and coated with PBAE/NaAIg layers using extrusion and layer-by-layer processes. The liposomes were prepared by lipids film hydration method, followed by loading drug through the pH-gradient method. Then, the positively charged PBAE and negatively charged NaAIg layers were coated on the surface *via* a layer-by-layer process. These Spe@HNPs can passively deposit at the infection site and release the drug by responding to the acid at the infectious microenvironment after intravenous administration, followed by eliminating the bacteria and treating the mouse lung infection (ALI). Spe@HNPs can effectively treat the drug-resistant bacterium-induced ALI compared with free drugs. The reason might be that Spe@HNPs with positive surface charge due to the protonation of tertiary amine residues in the PBAE layer under an acidic environment could break the bacterial cell wall, induce the death of bacteria and exhibit the synergetic effect with the drug. This work not only reports a promising nanomedicine for ALI treatment but also provides an effective approach to fabricate a multi-functional multi-layers nanosystem for drug delivery and controlled release. The mechanism of synergetic effect from polycationic polymer-based carrier and antibiotic is very important for the development of therapeutics for antimicrobial resistance. And we would be focused on this in the future.

## References

[CIT0001] Arias CA, Murray BE. (2015). A new antibiotic and the evolution of resistance. N Engl J Med 372:1168–70. 25785976 10.1056/NEJMcibr1500292 PMC4433155

[CIT0002] Aslam B, Wang W, Arshad MI, et al. (2018). Antibiotic resistance: a rundown of a global crisis. Infect Drug Resist 11:1645–58. 30349322 10.2147/IDR.S173867 PMC6188119

[CIT0003] Ayukekbong JA, Ntemgwa M, Atabe AN. (2017). The threat of antimicrobial resistance in developing countries: causes and control strategies. Antimicrob Resist Infect Control 6:47. 28515903 10.1186/s13756-017-0208-x PMC5433038

[CIT0004] Blaskovich MAT. (2018). The fight against antimicrobial resistance is confounded by a global increase in antibiotic usage. ACS Infect Dis 4:868–70. 29757608 10.1021/acsinfecdis.8b00109

[CIT0005] Chen X, Guo R, Wang C, et al. (2021). On-demand pH-sensitive surface charge-switchable polymeric micelles for targeting *Pseudomonas aeruginosa* biofilms development. J Nanobiotechnology 19:99 33836750 10.1186/s12951-021-00845-0 PMC8034112

[CIT0006] Deng ZJ, Morton SW, Ben-Akiva E, et al. (2013). Layer-by-layer nanoparticles for systemic codelivery of an anticancer drug and siRNA for potential triple-negative breast cancer treatment. ACS Nano 7:9571–84. 24144228 10.1021/nn4047925 PMC3870477

[CIT0007] Deshmukh PK, Ramani KP, Singh SS, et al. (2013). Stimuli-sensitive layer-by-layer (LbL) self-assembly systems: targeting and biosensory applications. J Control Release 166:294–306. 23313111 10.1016/j.jconrel.2012.12.033

[CIT0008] Dinarello CA. (2010). Anti-inflammatory agents: present and future. Cell 140:935–50. 20303881 10.1016/j.cell.2010.02.043 PMC3752337

[CIT0009] Tacconelli F, Sifakis S, Harbarth R, et al. (2018). Surveillance for control of antimicrobial resistance. Lancet Infect Dis 18:e99–e106. 29102325 10.1016/S1473-3099(17)30485-1

[CIT0010] Freag MS, Elnaggar YS, Abdelmonsif DA, Abdallah OY. (2016). Layer-by-layer-coated lyotropic liquid crystalline nanoparticles for active tumor targeting of rapamycin. Nanomedicine 11:2975–96. 27785978 10.2217/nnm-2016-0236

[CIT0011] Gupta A, Mumtaz S, Li C-H, et al. (2019). Combatting antibiotic-resistant bacteria using nanomaterials. Chem Soc Rev 48:415–27. 30462112 10.1039/c7cs00748e PMC6340759

[CIT0012] Holmes AH, Moore LSP, Sundsfjord A, et al. (2016). Understanding the mechanisms and drivers of antimicrobial resistance. Lancet 387:176–87. 26603922 10.1016/S0140-6736(15)00473-0

[CIT0013] Huang X, Liao W, Zhang G, et al. (2017). pH-sensitive micelles self-assembled from polymer brush (PAE-g-cholesterol)-b-PEG-b-(PAE-g-cholesterol) for anticancer drug delivery and controlled release. Int J Nanomedicine 12:2215–26. 28356738 10.2147/IJN.S130037 PMC5367585

[CIT0014] Hussain S, Joo J, Kang J, et al. (2018). Antibiotic-loaded nanoparticles targeted to the site of infection enhance antibacterial efficacy. Nat Biomed Eng 2:95–103. 29955439 10.1038/s41551-017-0187-5 PMC6015743

[CIT0015] Ilgin P, Ozay H, Ozay O. (2020). Synthesis and characterization of pH responsive alginate based-hydrogels as oral drug delivery carrier. J Polym Res 27:251.

[CIT0016] Jain D, Bar-Shalom D. (2014). Alginate drug delivery systems: application in context of pharmaceutical and biomedical research. Drug Dev Ind Pharm 40:1576–84. 25109399 10.3109/03639045.2014.917657

[CIT0017] Kaczmarek JC, Patel AK, Kauffman KJ, et al. (2016). Polymer–lipid nanoparticles for systemic delivery of mRNA to the lungs. Angew Chem 128:14012–6. 10.1002/anie.201608450 PMC5279893 27690187

[CIT0018] Kavanagh KT. (2019). Control of MSSA and MRSA in the United States: protocols, policies, risk adjustment and excuses. Antimicrob Resist Infect Control 8:103. 31244994 10.1186/s13756-019-0550-2 PMC6582558

[CIT0019] Klein E, Smith DL, Laxminarayan R. (2007). Hospitalizations and deaths caused by methicillin-resistant *Staphylococcus aureus*, United States. Emerg Infect Dis 13:1840–6. 18258033 10.3201/eid1312.070629 PMC2876761

[CIT0020] Kuno T, Hirayama-Kurogi M, Ito S, Ohtsuki S. (2016). Effect of intestinal flora on protein expression of drug-metabolizing enzymes and transporters in the liver and kidney of germ-free and antibiotics-treated mice. Mol Pharm 13:2691–701. 27376980 10.1021/acs.molpharmaceut.6b00259

[CIT0021] Lee RE, Hurdle JG, Liu J, et al. (2014). Spectinamides: a new class of semisynthetic antituberculosis agents that overcome native drug efflux. Nature Medicine 20:152–8. 10.1038/nm.3458 PMC3972818 24464186

[CIT0022] Li J, Ma YJ, Wang Y, et al. (2018). Dual redox/pH-responsive hybrid polymer-lipid composites: Synthesis, preparation, characterization and application in drug delivery with enhanced therapeutic efficacy. Chem Eng J 341:450–61.

[CIT0023] Liao S, Zhang Y, Pan X, et al. (2019). Antibacterial activity and mechanism of silver nanoparticles against multidrug-resistant Pseudomonas aeruginosa. IJN 14:1469–87. 30880959 10.2147/IJN.S191340 PMC6396885

[CIT0024] Ling LL, Schneider T, Peoples AJ, et al. (2015). A new antibiotic kills pathogens without detectable resistance. Nature 517:455–9. 25561178 10.1038/nature14098 PMC7414797

[CIT0025] Ma Z, Li J, Bai Y, et al. (2020). A bacterial infection-microenvironment activated nanoplatform based on spiropyran-conjugated glycoclusters for imaging and eliminating of the biofilm. Chemical Engineering Journal 399:125787.

[CIT0026] Mehta D, Malik AB. (2006). Signaling mechanisms regulating endothelial permeability. Physiol Rev 86:279–367. 16371600 10.1152/physrev.00012.2005

[CIT0027] Men W, Zhu P, Dong S, et al. (2020). Layer-by-layer pH-sensitive nanoparticles for drug delivery and controlled release with improved therapeutic efficacy in vivo. Drug Deliv 27:180–90. 31924103 10.1080/10717544.2019.1709922 PMC7008239

[CIT0028] Mendy A, Vieira ER, Albatineh AN, Gasana J. (2016). *Staphylococcus aureus* colonization and long-term risk for death, United States. Emerg Infect Dis 22:1966–9. 27767920 10.3201/eid2211.160220 PMC5088013

[CIT0029] Mensah LB, Morton SW, Li J, et al. (2019). Layer-by-layer nanoparticles for novel delivery of cisplatin and PARP inhibitors for platinum-based drug resistance therapy in ovarian cancer. Bioeng Transl Med 4:e10131. 31249881 10.1002/btm2.10131 PMC6584097

[CIT0030] Molinaro R, Corbo C, Martinez JO, et al. (2016). Biomimetic proteolipid vesicles for targeting inflamed tissues. Nat Mater 15:1037–46. 27213956 10.1038/nmat4644 PMC5127392

[CIT0031] Morton SW, Poon Z, Hammond PT. (2013). The architecture and biological performance of drug-loaded LbL nanoparticles. Biomaterials 34:5328–35. 23618629 10.1016/j.biomaterials.2013.03.059 PMC4040352

[CIT0032] Prestinaci F, Pezzotti P, Pantosti A. (2015). Antimicrobial resistance: a global multifaceted phenomenon. Pathog Glob Health 109:309–18. 26343252 10.1179/2047773215Y.0000000030 PMC4768623

[CIT0033] Radovic-Moreno AF, Lu TK, Puscasu VA, et al. (2012). Surface charge-switching polymeric nanoparticles for bacterial cell wall-targeted delivery of antibiotics. ACS Nano 6:4279–87. 22471841 10.1021/nn3008383 PMC3779925

[CIT0034] Klevens MA, Morrison J, Nadle S, et al. (2007). Invasive methicillin-resistant *Staphylococcus aureus* infections in the United States. JAMA 298:1763–71. 17940231 10.1001/jama.298.15.1763

[CIT0035] Roberts DM. (2011). The relevance of drug clearance to antibiotic dosing in critically ill patients. Curr Pharm Biotechnol 12:2002–14. 21554217 10.2174/138920111798808374

[CIT0036] Si Z, Hou Z, Vikhe YS, et al. (2021). Antimicrobial effect of a novel chitosan derivative and its synergistic effect with antibiotics. ACS Appl Mater Interfaces 13:3237–45. 33405504 10.1021/acsami.0c20881

[CIT0037] Thanner S, Drissner D, Walsh F. (2016). Antimicrobial resistance in agriculture. mBio 7:e02227–e02215. 27094336 10.1128/mBio.02227-15 PMC4850276

[CIT0038] van der Poll T, van de Veerdonk FL, Scicluna BP, Netea MG. (2017). The immunopathology of sepsis and potential therapeutic targets. Nat Rev Immunol 17:407–20. 28436424 10.1038/nri.2017.36

[CIT0039] Wade S, Williams M. (2019). Antibiotic side‐effects: from the anticipated to the bizarre. Prescriber 30:16–21.

[CIT0040] Yang Y, Ding Y, Fan B, et al. (2020). Inflammation-targeting polymeric nanoparticles deliver sparfloxacin and tacrolimus for combating acute lung sepsis. J Control Release 321:463–74. 32087302 10.1016/j.jconrel.2020.02.030

[CIT0041] Zhang CY, Gao J, Wang Z. (2018). Bioresponsive nanoparticles targeted to infectious microenvironments for sepsis management. Adv Mater 30:1803618. 10.1002/adma.201803618 PMC6197919 30203430

[CIT0042] Zhang CY, Lin W, Gao J, et al. (2019). pH-responsive nanoparticles targeted to lungs for improved therapy of acute lung inflammation/injury. ACS Appl Mater Interfaces 11:16380–90. 30973702 10.1021/acsami.9b04051 PMC6542597

[CIT0043] Zhang CY, Xiong D, Sun Y, et al. (2014). Self-assembled micelles based on pH-sensitive PAE-g-MPEG-cholesterol block copolymer for anticancer drug delivery. Int J Nanomedicine 9:4923–33. 25364250 10.2147/IJN.S69493 PMC4211906

